# A new application of hydrocolloids from *Echinops setifer* (Shekartighal): Mayonnaise‐type sauces

**DOI:** 10.1002/fsn3.3881

**Published:** 2023-11-30

**Authors:** Narges Nemati, Mohammad Ali Hesarinejad

**Affiliations:** ^1^ Department of Food Processing Research Institute of Food Science and Technology (RIFST) Mashhad Iran

**Keywords:** antioxidant, enrichment, functional food, mayonnaise‐type sauce, Shekartighal

## Abstract

Due to the importance of the production of functional and nutraceutical foods with high added value, Shekartighal was used to develop ready‐made functional mayonnaise‐type sauces and evaluate their physicochemical, sensory, and antioxidant properties over 60 days of storage. Shekartighal had, on average, 76.85% carbohydrate, 9.24% protein, 4.91% fat, 1.74% ash, 7.26% moisture, 20.38 mgGAE/g total phenol content, 14.31 μM ferrous sulphate/g, and 145.12 μg/mL antioxidant activity. The results indicated that Shekartighal had high water absorption capacity (408.01%) and emulsion stability (91.83%). Following the textural and sensorial study, the product that stood out as the best performer was the sauce containing 3% Shekartighal. Consequently, this product was selected for further evaluation, where its physicochemical and antioxidant properties were assessed for a 60‐day duration under refrigerated storage (4°C). The formulation with 3% Shekartighal presented significantly more phenolic content and antioxidant properties than the control. During the 60‐day refrigerated storage (4°C) period, the physicochemical and antioxidant parameters of the enriched sauce remained stable. The addition of Shekartighal to the sauce significantly improved its nutritional quality, and the sauce enriched with 3% Shekartighal demonstrated the most favorable characteristics among the different concentrations tested. Overall, these findings suggest that Shekartighal could be utilized as a natural ingredient to create a functional sauce with notable antioxidant and nutritional properties.

## Graphical abstract

This study developed functional mayonnaise‐type sauces using Shekartighal and evaluated their physicochemical, sensory, and antioxidant properties over 60 days of storage. The results showed that the sauce containing 3% Shekartighal had high water absorption capacity and emulsion stability and presented significantly more phenolic content and antioxidant properties than the control. The addition of Shekartighal improved the nutritional quality of the sauce, suggesting that it could be used as a natural ingredient to create a functional sauce with notable antioxidant and nutritional properties.

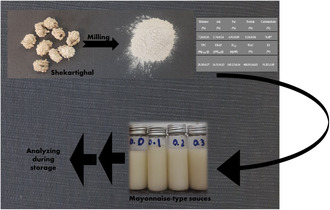

## 1 INTRODUCTION

*Echinops*, a genus containing about 120–130 species in the Compositae family, is seen worldwide, especially in the Mediterranean, Africa, and Central Asia (Benvidi & Jahanbin, 2020; Sánchez‐Jiménez et al., 2010). This hydrocolloid, which is known in Persian as ShekarTighal, is almost obtained as a result of the activity of weevils of the genus *Larinus* with the scientific name *Larinus vulpes* (Olivier, 1807) (Curculionidae: Lixinae: *Lixini*) on some species of the genus *Echinops* (Khameh et al., 2020). Only six *Larinus* species construct analogous capsules (pupal chambers) with a sweet taste (known as “trehala”) on these plant stems (Skuhrovec et al., 2017). In many areas, despite the abundance of this plant, there are no traces of the activity and presence of the insect (Nasirzadeh et al., 2005). This substance has a sweet and glazed taste that has laxative and analgesic properties and has been widely used in traditional medicine since ancient times (Hamedi et al., 2015). Recent research indicates that Shekartighal contains numerous bioactive compounds, including flavonoids, polysaccharides, and terpenoids that possess antimicrobial, antioxidant, and anti‐inflammatory properties (Bitew & Hymete, 2019; Hamedi et al., 2015; Mohammadi & Dini, 2003; Soori et al., 2019; Tavakoli et al., 1999).

The use of medicinal plants in fortifying mayonnaise‐type sauces is an emerging research area due to the potential health benefits from their bioactive compounds, such as polyphenols, flavonoids, and terpenoids, which have antioxidant, anti‐inflammatory, and antimicrobial properties. These compounds can be added to enhance the nutritional profile and health‐promoting properties of the sauce (Mirzanajafi‐Zanjani et al., 2019; Wijayanti et al., 2023). The inclusion of *Rosmarinus officinalis* L. extract, which is abundant in polyphenols, has been demonstrated to enhance the antioxidant activity of mayonnaise‐type sauces (Alizadeh et al., 2019), while the incorporation of ginger extract has been found to boost the anti‐inflammatory features of such sauces (Kishk & Elsheshetawy, 2013). Additionally, various herbs, spices, and flavorings can be added to enhance the taste and aroma of the sauce (Thomas et al., 2021). Careful selection of the ingredients and their appropriate proportions is essential to achieve an enriched mayonnaise‐type sauce that is both nutritious and palatable.

Polysaccharides have been studied for their potential application in the fortification of mayonnaise‐type sauces. Polysaccharides are complex carbohydrates that can be derived from various sources such as plants, animals, and microorganisms (Eghbaljoo et al., 2022; Ma & Boye, 2014). They have the ability to form gels, thicken solutions, and stabilize emulsions, which makes them suitable for use in the food industry. In mayonnaise‐type sauces, polysaccharides can be added to improve the texture, stability, and nutritional properties (Chivero et al., 2016). For example, the addition of pectin, a polysaccharide derived from fruits, has been shown to improve the viscosity and stability of mayonnaise‐type sauces (Chang et al., 2017). Nevertheless, determining the desired level of addition of both polysaccharides and medicinal plant extracts is crucial to prevent negative impacts on the sensory properties of the sauce. As mentioned, the enrichment of mayonnaise‐type sauces is a common practice to improve their nutritional value and sensory properties. Therefore, the aims of the present study were to use Shekartighal in mayonnaises as a preservative, antioxidant, and functional nutritional agent.

## 2 MATERIALS AND METHODS

### 2.1 Materials

Sunflower oil, mustard, pasteurized whole egg, vinegar, sugar, and salt were bought locally (Mashhad, Iran). Commercial xanthan gum (Food grade xanthan gum, Sigma‐Aldrich Co.) was used in this study. All other reagents were of analytical grade (Merck, Darmstadt, Germany). *Echinops setifer* was collected from the Saveh region (35°21′36″ North, 50°13′12″ East) from Iran. Shade‐dried Shekartighal was milled using an electrical blender (Parskhazar, Iran) and sieved through a mesh 18.

### 2.2 Methods

#### 2.2.1 Sauce formulations

In order to determine the specific ingredients used in the sauce for the study, initial tests were conducted using varying proportions of the ingredients and Shekartighal. The parameters used to assess the sauce included visual appearance, dominant spice aroma, consistency, evenness, and smooth texture. Four sauce formulations, according to the recipe described by Heggset et al. (2020) with some modifications containing 0 (control), 1.0, 2.0, and 3.0% Shekartighal (wt.%), sunflower oil (65.00 g), vinegar (5.40 g), pasteurized whole egg (10.00 g), mustard (0.50 g), garlic powder (0.50 g), sugar (1.00 g), sodium chloride (1.50 g), xanthan (0.10 g), and water (16.00 mL), were prepared. Initially, the Shekartighal powder was dissolved in distilled water at 60°C for 30 min with the use of a magnetic stirrer (IKA, Germany) to ensure complete hydration for the continuous phase. This solution was then combined with vinegar, salt, sugar, mustard, egg, and garlic powder in a high‐speed mixer (Kitchen Blender, Pars‐Khazar, Iran). In order to create the dispersed phase, oil was added to the solution that was previously prepared and homogenized in a rotor–stator system (Ultra‐Turrax T25 basic, IKA, Germany) for 5 min at 12,000 rpm. The freshly prepared mayonnaise‐type sauces were poured into sterilized glass jars and pasteurized by heating the jars for 40 s at 98°C (Pérez‐Conesa et al., 2009) and stored in refrigerator (4°C).

#### 2.2.2 Bromatological analysis

Moisture content was evaluated by dehydrating the samples in an oven at 105°C based on AOAC 33.7.03 Method 926.08. The fat content was measured using AOAC method 972.28, while ash content was determined using AOAC 33.7.07, and protein content was determined through the Kjeldahl method following AOAC 33.7.12 Method 926.123. All techniques used for the analysis were in accordance with the protocols established by the Association of Official Analytical Chemists.

The total carbohydrates were estimated using the difference, as shown in Equation (1).

(1) $$\text{Total carbohydrates}\;\left(\%\right)=100-\left(\text{Moisture}+\text{Total}\;\mathrm{ash}+\text{Total nitrogen}+\text{Total lipids}\right)$$

#### 2.2.3 Functional properties

The emulsion stability and water absorption capacity were determined using the method outlined in our previous study (Koocheki et al., 2012). Briefly, the sample was dispersed into deionized water and stored overnight at 4°C. Sunflower oil was slowly added into the dispersion and homogenized using Ultra‐Turrax T‐25 homogenizer (IKA Instruments, Germany) at 9000 rpm for 2 min. The stability of the emulsion against high temperature (85°C) was determined from the ratio of the final volume to the initial volume of the emulsion. The sample was completely wetted with distilled water and centrifuged at 1600 *g* for 10 min. The swollen sample was weighed and the difference in weight between the swollen sample and the initial sample was divided by the initial sample to determine the amount of water absorption capacity.

#### 2.2.4 Total phenolic content and antioxidant activity

The antioxidant activity of the samples was determined by measuring the activity of the radical scavenger DPPH and ferric ion‐reducing antioxidant power (FRAP) assay according to Koocheki et al. (2022). The total phenolic content (TPC) was assessed using the Folin–Ciocalteu technique. A calibration curve was created using the standard compound of gallic acid and measuring its absorbance at 750 nm as described by Behbahani et al. (2017).

#### 2.2.5 Texture analysis

The texture of samples that had been stored at a temperature of 4°C for 24 h was evaluated using a texture analyzer (CT3 Texture Analyzer; Brookfield, The USA). The samples underwent double‐cycle compression to 50% of their original thickness at a test speed of 0.5 mm s^−1^ and 5 g trigger load. Firmness (N), cohesiveness, and elasticity (mm) were among the texture profile parameters that were assessed.

#### 2.2.6 Sensory evaluation

A total of 40 untrained panelists, comprising 55% women and 45% men, aged between 18 and 40 years, were recruited to conduct the sensory evaluation (Hough et al., 2006). The panelists were presented with four mayonnaise‐type sauce samples, including the control formulation and the formulations with varying concentrations of Shekartighal. The samples were randomly assigned three‐digit numbers and served on small white plates with disposable spoons. To taste the sauce, the panelists were provided with 25‐g portions of bread in disposable containers. The panelists evaluated the formulations' overall acceptability using a 9‐point hedonic scale, ranging from 1 (dislike extremely) to 9 (like extremely), based on attributes such as appearance, aroma, flavor, texture, and overall impression. Additionally, the panelists were asked about their purchase intent on a scale from 1 (definitely would not buy) to 4 (definitely would buy) (Almeida et al., 2021).

#### 2.2.7 Droplet size measurement

The droplet size average was computed utilizing a particle size analyzer (Cordouan, French, NanoQ software Vasco 3) based on laser light scattering at room temperature (20 ± 1°C).

### 2.3 Statistical analysis

The mean values ± standard deviations of the analyses, performed in triplicate, were reported. ANOVA and Tukey's test (*p* < .05) were applied to compare the differences between the control and mayonnaise‐type formulations containing 0%, 1%, 2%, and 3% of Shekartighal. The formulation with the best physicochemical parameters and sensory attributes was selected for further evaluation during 60 days of storage. The Student's *t*‐test was used to compare the control with the selected formulation at days 0 and 60 of storage.

## 3 RESULTS AND DISCUSSIONS

### 3.1 Characterization and functional properties of Shekartighal

Table 1 provides information on the chemical composition and antioxidant activity of Shekartighal. Chemical analysis indicated that Shekartighal contained 9.24% protein, 7.26% moisture, 1.74% ash, and 4.91% fat. Comparing our results to the other natural mucilage extracted indicated different chemical compositions. Although the protein content was higher and carbohydrate content was lower than those reported for *L. perfoliatum* and *Ocimum basilicum* by Koocheki et al. (2013) and Hosseini‐Parvar et al. (2010), respectively, the ash and moisture contents were lower than those reported for *Eruca sativa* by Koocheki et al. (2012).

**TABLE 1:** Characterization of Shekartighal.

Moisture (%)	Ash (%)	Fat (%)	Protein (%)	Carbohydrate (%)	TPC (mg _GAL_/g)	FRAP (μM_FeII_/g)	IC_50_ (μg/mL)	WAC (%)	ES (%)
7.26 ± 0.26	1.74 ± 0.24	4.91 ± 0.05	9.24 ± 0.56	76.85^a^	20.38 ± 0.37	14.31 ± 0.13	145.12 ± 0.14	408.01 ± 16.03	91.83 ± 3.05

^a^ Total carbohydrate content was subtracted from 100% − (Moisture + Ash + Fat + Protein).

The FRAP assay employs the Fe^2+^ concentration in samples to detect the presence of antioxidants and evaluate their potential for reducing ability (Koocheki et al., 2022). The results revealed that Shekartighal's TPC and FRAP were 20.38 ± 0.37 and 14.31 ± 0.13, respectively. These values were lower than those reported for *Plantago major* gum, which had TPC and FRAP values of 76.79 ± 1.6 and 97.80 ± 2.00, respectively (Behbahani et al., 2017). The DPPH test was used to determine the free radical scavenging activity of Shekartighal. The extract concentration that scavenged 50% of free radicals (IC50) was calculated to be 145.12 ± 0.14 μg/mL. The antioxidant activity of Shekartighal was similar to that reported for Flixweed seeds mucilage (40–360) by Golalikhani et al. (2014), but lower than the value reported for *Lepidium perfoliatum* seed gum (4300) by Koocheki et al. (2022).

Table 1 also provides information on some functional properties of Shekartighal, such as emulsion stability (ES) and water absorption capacity (WAC). Polysaccharides have been found to have a significant impact on the stability of o/w emulsions concerning flocculation, coalescence, and creaming. According to previous research by Dickinson (2003), hydrocolloids stabilize emulsions by altering the rheological properties of the aqueous phase, which prevents the dispersed‐phase droplets from interacting with each other and resulting in destabilization processes such as coalescence and flocculation. This study demonstrated that Shekartighal, as a novel hydrocolloid, had a significant emulsion stabilizing effect (91.83%).

The water absorption capacity (WAC) of different hydrocolloid sources can be attributed to the level of polar hydroxyl groups in the samples, which affects their degree of hydrodynamic interaction (Koocheki et al., 2013). Hydrocolloids can absorb significant amounts of water due to the unfolding of their constituent polysaccharides and their higher branching proportion, as noted by Sciarini et al. (2009). Shekartighal exhibited a high WAC value (408.01%), although it was much lower than that reported for other hydrocolloids such as *Eruca sativa* (13.91 g/g) as reported by Koocheki et al. (2012).

### 3.2 Preliminary results of mayonnaise‐type sauces

#### 3.2.1 Physicochemical analysis

In this study, the effect of Shekartighal supplementation on the pH and acidity of sauces was investigated. The control formulation had lower pH and higher acidity compared to the formulations with Shekartighal. The pH of the sauces increased while the acidity decreased with the addition of Shekartighal, which was attributed to the neutral or slightly basic pH of Shekartighal (≈8). Total acidity is an essential parameter for the preservation and acceptance of food products, similar to pH. The hydrogen ion concentration and acidity of food products can be affected by various processes such as hydrolysis, oxidation, or fermentation, leading to decomposition (Isildak & Gones, 2018).

Total acidity values also differed among the formulations, with the highest value observed in the control sample. A negative correlation between pH and acidity was observed, where lower pH resulted in higher acidity. The formulation with 3% Shekartighal had the lowest acidity value, which was attributed to the alkaline character of Shekartighal. These findings highlight the potential of Shekartighal as a natural ingredient to modify the pH and acidity of food products.

#### 3.2.2 Texture analysis

Texture parameters such as firmness, cohesiveness, and elasticity are critical food attributes and are correlated with sensory properties (Talens et al., 2017). In this study, the texture profile and sensory parameters of formulations containing different concentrations of Shekartighal were evaluated. The firmness, cohesiveness, and elasticity values significantly varied between the control and formulations with 1%, 2%, and 3% Shekartighal (*p* < .05). The addition of Shekartighal changed the texture profile of the sauces, resulting in firmer and more elastic products with increasing concentrations of Shekartighal. The formulation with 3% Shekartighal showed the highest firmness value (Table 2). The addition of Shekartighal to the sauces led to notable variations in cohesiveness and elasticity across all treatments (*p* < .05). In particular, the sauce that included 3% Shekartighal exhibited the highest elasticity. The findings appear to be associated with the carbohydrate composition of Shekartighal.

**TABLE 2:** pH, acidity, textural parameters, and sensory characteristics of the mayonnaise‐type sauces supplemented with different Shekartighal concentrations after 24 h of storage.

Parameters	Control sample	Shekartighal concentrations (%)		
		1	2	3
pH	3.15 ± 0.03^d^	3.24 ± 0.06^c^	3.71 ± 0.03^b^	4.05 ± 0.05^a^
Total acidity (%)	3.08 ± 0.05^a^	2.98 ± 0.09^b^	2.23 ± 0.07^c^	1.94 ± 0.09^d^
Textural parameters				
Firmness (N)	0.47 ± 0.01^b^	0.49 ± 0.03^b^	0.56 ± 0.02^a^	0.58 ± 0.04^a^
Cohesiveness	2.40 ± 0.13^a^	2.48 ± 0.07^a^	2.53 ± 0.17^a^	2.54 ± 0.02^a^
Elasticity (nm)	3.51 ± 0.39^d^	4.70 ± 0.25^c^	4.91 ± 0.40^b^	5.42 ± 0.21^a^
Sensory parameters				
Appearance	7.85 ± 1.21^a^	7.04 ± 0.97^ab^	6.60 ± 0.89^ab^	6.12 ± 1.70^b^
Aroma	7.13 ± 0.50^a^	7.24 ± 0.71^a^	6.96 ± 0.63^a^	7.02 ± 0.71^a^
Flavor	6.72 ± 0.83^a^	6.87 ± 0.78^a^	6.46 ± 0.50^a^	6.50 ± 0.81^a^
Texture	7.85 ± 0.20^a^	7.64 ± 0.61^a^	7.96 ± 1.12^a^	7.32 ± 1.71^a^
Overall impression	6.90 ± 1.18^a^	6.76 ± 1.73^a^	6.59 ± 1.60^a^	6.74 ± 1.51^a^
Purchase intent	2.89 ± 0.93^a^	2.78 ± 0.97^a^	2.83 ± 1.07^a^	2.97 ± 0.92^a^

*Note*: Different lowercase letters on the same line indicate a significant difference between the sauces analyzed (*p* < .05).

#### 3.2.3 Sensory evaluation

The three important organoleptic attributes of food products are texture, appearance, and flavor, which collectively determine consumer acceptability. The sensory evaluation indicated that the use of Shekartighal had no impact on the panelists' acceptance of the product. The addition of 1%, 2%, and 3% Shekartighal to the sauces did not have any significant effect on the product's aroma, flavor, texture, or overall impression purchase (*p* < .05). It seems that the spices' blended aroma employed in making the sauces played a crucial role in masking any possible unpleasant taste. The unpleasant aroma was effectively covered up in the sauce by sulfur‐containing compounds like iso‐allin, methiin, and propiin found in onions (Cecchi et al., 2020) and allicin in garlic (Abe et al., 2020). Additionally, cumin (Kaban, 2013), rosemary (Tomi et al., 2016), parsley (Farouk et al., 2017), and oregano (Bansleben et al., 2009) also contributed to masking the undesirable odor in the food. It is important to note that the sauce had a significant amount of spices, and their fragrances were easily detectable by the olfactory system.

The inclusion of Shekartighal caused a noticeable change in the color of the products, resulting in a yellowish appearance. As a result, the appearance scores (Table 2) indicated that the panelists perceived the color as unsatisfactory. This perception could be attributed to the fact that the panelists were not adequately trained. Consequently, the control sauce scored significantly higher (*p* < .05) in the appearance attribute than the sauces that were supplemented with 2% and 3% Shekartighal. Despite the difference in color, the purchase intent did not differ significantly (*p* > .05) between the control sauce and the sauces that contained Shekartighal (Table 2). The score for this attribute was ≈3.0 on the hedonic scale, indicating that the panelists “would probably buy” the product. Although not statistically significant, the sauce that contained 3% Shekartighal received the highest score (2.97), with 69.6% of the panelists indicating that they would buy it. Furthermore, the aroma, flavor, and texture remained unchanged after the addition of 1%, 2%, and 3% Shekartighal, indicating general acceptability among panelists.

The sauce that contained 3% Shekartighal received the highest scores for purchase intent and overall impression, suggesting that the appearance did not have a negative impact on these attributes. Moreover, the sauce that contained 3% Shekartighal exhibited optimal physical characteristics such as pH, acidity, and texture, which are essential for ensuring the final product's quality. It is expected that the formulations with higher levels of Shekartighal incorporation will have higher nutritional values due to the proximate composition of Shekartighal. The sauce that contained 3% Shekartighal was identified as the top‐performing product in this study. As a result, it was chosen for additional analyses to assess its physicochemical and antioxidant properties during a 60‐day refrigerated storage period (4°C).

### 3.3 Average droplet size

Table 3 indicates that the addition of Shekartighal to the mayonnaise‐type sauce resulted in smaller droplet sizes, with the sauce containing 3% Shekartighal showing a lower droplet size than control sample on the first day of production. The mayonnaise‐type sauce sample containing 3% Shekartighal had an average droplet size of 0.63 μm, which was significantly lower than that of the control sample, which had an average droplet size of 1.35 μm. This smaller droplet size obtained with Shekartighal prevented oil droplet coalescence and maintained the product's stability (Mousakhani‐Ganjeh & Goli, 2021). The droplet size of the samples did not change during storage for up to 60 days in the case of the sauce containing 3% Shekartighal and up to 30 days for the control sample. The addition of Shekartighal to the mayonnaise formula decreased the average droplet size. The stabilizers directly modified the rheological properties, continually increasing the viscosity in the continuous phase. This viscosity increase reduced the movement of the oil droplets toward each other, preventing them from bonding to form larger droplets as reported by Golkar, Nasirpour and Keramat (2015), Golkar, Nasirpour, Keramat and Desobry (2015) and Puligundla et al. (2015). Additionally, the physical barrier role of hydrocolloids prevents droplet flocculation and coalescence after emulsion preparation, resulting in smaller particle sizes for the mayonnaise samples.

**TABLE 3:** Variations in the average droplet size, total phenolic content, and antioxidant activity in the control sauce and the sauce with 3% Shekartighal stored for 60 days at 4°C.

Parameter	Sample	Time (day)		
		0	30	60
Average droplet size (μm)	Control	1.35 ± 0.06^aA^	1.30 ± 0.03^aA^	1.23 ± 0.04^bA^
	3% Shekartighal	0.63 ± 0.02^aB^	0.61 ± 0.01^aB^	0.59 ± 0.03^aB^
TPC (mg _GAL_/g)	Control	120.12 ± 0.85^aB^	109.87 ± 0.75^bB^	108.65 ± 0.21^bB^
	3% Shekartighal	190.41 ± 0.49^aA^	187.07 ± 0.34^abA^	185.77 ± 0.39^bA^
FRAP (μmol_FeII_/g)	Control	230.46 ± 0.80^aB^	226.88 ± 0.25^bB^	223.12 ± 0.41^cB^
	3% Shekartighal	312.05 ± 0.75^aA^	310.45 ± 0.39^abA^	308.80 ± 0.32^bA^
IC_50_ (μg/mL)	Control	186.12 ± 0.66^bA^	188.09 ± 0.21^abA^	190.09 ± 0.24^aA^
	3% Shekartighal	89.32 ± 0.50^bB^	89.80 ± 0.46^abB^	90.14 ± 0.23^aB^

*Note*: The data are presented as the mean ± standard deviation of triplicates. Lowercase letters within the same line indicate a significant difference between the means of the same sauce (control or 3% Shekartighal supplemented sauce) at different times as determined by the Tukey's test (*α* = .05). Capital letters within the same column indicate a significant difference between the control sauce and the 3% Shekartighal‐supplemented sauce, as determined by the *t*‐test (*α* = .05).

### 3.4 Antioxidant properties

Food processing and storage can alter the antioxidant capacity of foods. Factors such as light, pH, temperature, oxygen exposure, and the presence of oxidizing enzymes can cause changes in the antioxidant activity, particularly when plant tissues are crushed or homogenized using various methods (Phillips et al., 2010). Additionally, the compounds that possess antioxidant properties may not react consistently to different radical or oxidant sources in a complex food system. Therefore, it is essential to use multiple assays to accurately assess the antioxidant capacities of foods (Ávila et al., 2019). The bioactive compound composition and antioxidant activity of the sauces were assessed using a set of methods, and the results are shown in Table 3. The sauce containing 3% Shekartighal had a higher total phenolic content than the control sauce (*p* < .05). The DPPH radical scavenging capacities and ferric reducing ability (FRAP) of the supplemented sauce were also higher than those of the control sauce during storage (*p* < .05). The sauce containing 3% Shekartighal had lower IC50 values than the control sauce, which remained approximately constant during storage. The increased antioxidant activity of the sauce containing Shekartighal can be attributed to the free radical scavenging activity of its phenolic compounds through hydrogen donation. After storage, the total phenolic content decreased by approximately ≈2% in the sauce containing Shekartighal and ≈9% in the control sauce. Similarly, the FRAP assay showed that the sauce supplemented with 3% Shekartighal had significantly higher antioxidant activity values than the control sauce (*p* < .05). While the control sauce also exhibited some antioxidant activity, it was lower than that of the sauce containing Shekartighal, indicating that the addition of Shekartighal significantly increased the sauce's antioxidant capacity. It is possible that the bioactive compounds in the ingredients used in both sauces (spices and oil) contributed to the control sauce's antioxidant activity.

## 4 CONCLUSION

The consumption of functional foods has been on the rise in recent years, offering consumers high nutritional value and healthier ingredients. The growing demand for sauces has also led to an increase in the variety of sauce formulations in the industry. This study investigated the use of Shekartighal in sauces at different concentrations (1%, 2%, and 3%) and found that it was well accepted by consumers due to its sensory properties. The formulations developed in this study met the current consumer demand for healthy and convenient foods. In addition, the results suggested that the sauce fortified with 3% Shekartighal had high phenolic compound and antioxidant potential, providing potential health benefits. Compared to the control formulation, the 3% Shekartighal fortified sauce showed superior properties. Therefore, it may be a suitable option for improving the dietary intake of individuals who regularly consume processed and easily prepared foods. Overall, this study demonstrated that Shekartighal, with its favorable antioxidant properties, can serve as a desirable natural additive in the manufacture of beneficial products.

## AUTHOR CONTRIBUTIONS

**Narges Nemati:** Conceptualization (equal); investigation (equal); software (equal); writing – original draft (equal). **Mohammad Ali Hesarinejad:** Conceptualization (equal); formal analysis (equal); methodology (equal); project administration (equal); software (equal); validation (equal); writing – original draft (equal); writing – review and editing (equal).

## FUNDING INFORMATION

Many thanks go to the Research Institute of Food Science and Technology in Mashhad for facilitating the process to conduct this study and providing laboratory equipment for experiments of this research.

## CONFLICT OF INTEREST STATEMENT

The authors declare no conflicts of interest.

## ETHICS STATEMENT

All participants gave informed consent before enrolling in the study, and all procedures were performed in accordance with ethical standards.

## CONSENT FOR PUBLICATION

All authors have read and agreed to the published version of the manuscript. All authors read and approved the final manuscript.

## Data Availability

All data generated or analyzed during this study are included in this published article.
